# Supplementary material for: Coupled epidemiological and wastewater modeling at the urban scale: A case study for Munich
Source: iScience. 2026 Jul 22;29(8):116891. doi: 10.1016/j.isci.2026.116891 (PMC13427414; doi:10.1016/j.isci.2026.116891)
Supplement: Document S1. Figures S1–S17 and Tables S1–S4 [file mmc1.pdf]

iScience, Volume *29*

Supplemental information

Coupled epidemiological and wastewater modeling at the urban scale: A case study for Munich

Julia Bicker, Natalie Tomza, Karina Wallrafen-Sam, Nina Schmid, Andreas F. Hofmann, Sascha Korf, Alain Schengen, Jasmin Javanmardi, Andreas Wieser, Martin J. Kühn, and Jan Hasenauer

A Supplementary figures

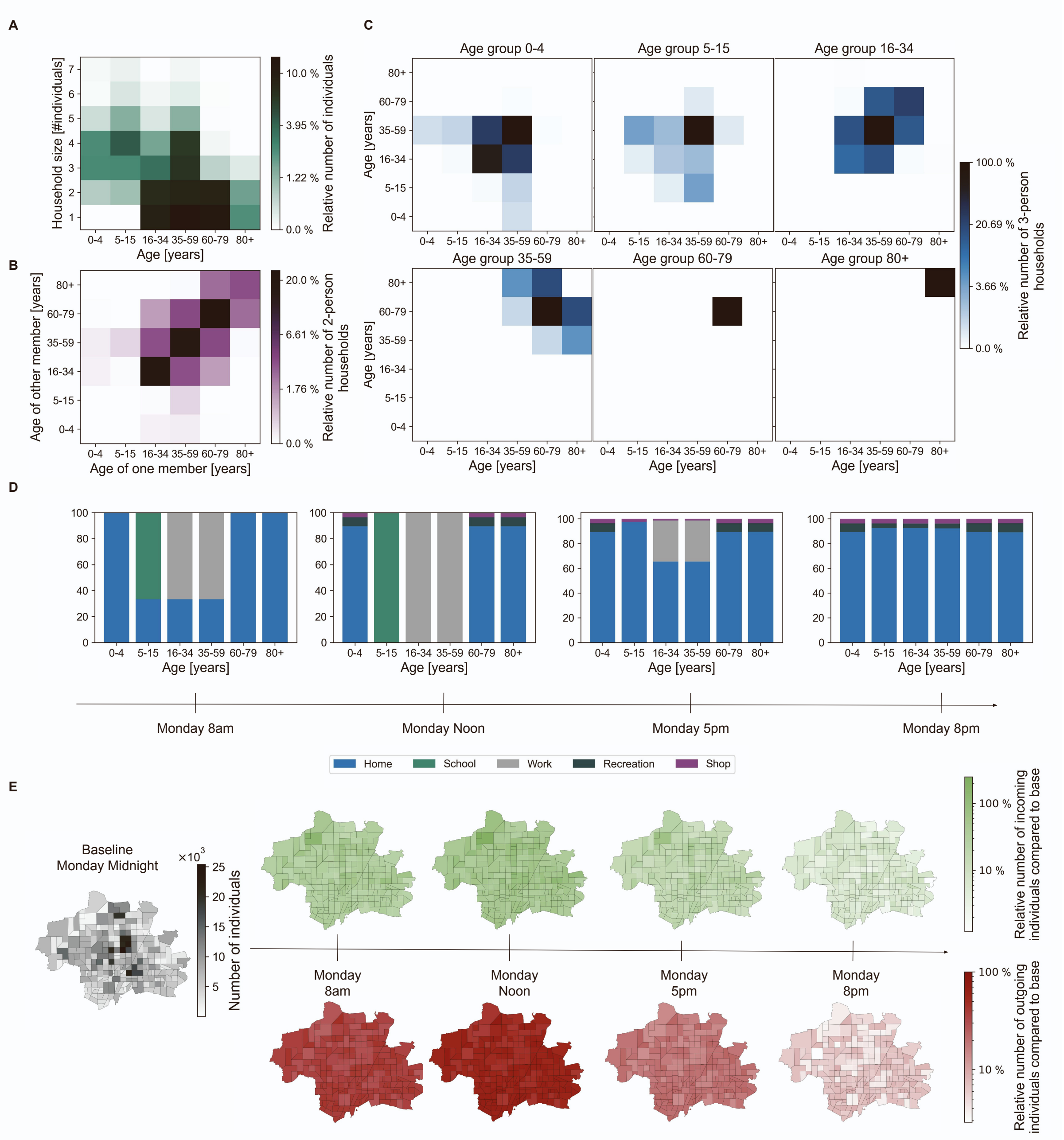

Supplementary Figure S1: ABM details, related to Figure 2. (A) Age structure of the modeled population across household sizes. **(B)** Age distribution of 2-person households. **(C)** Age distribution of 3-person households conditional on the age of the youngest household member. **(D)** Evolution of location type distributions during the day resolved by age group. Age group 5-15 is school-age while age groups 16-34 and 35-59 are working-age. **(E)** Movement of agents between wastewater areas throughout the day. Shown are the relative differences in the number of agents, separated by incoming (non-residents) and outgoing agents (residents), compared to the baseline of Monday at midnight, when all agents are at *Home*.

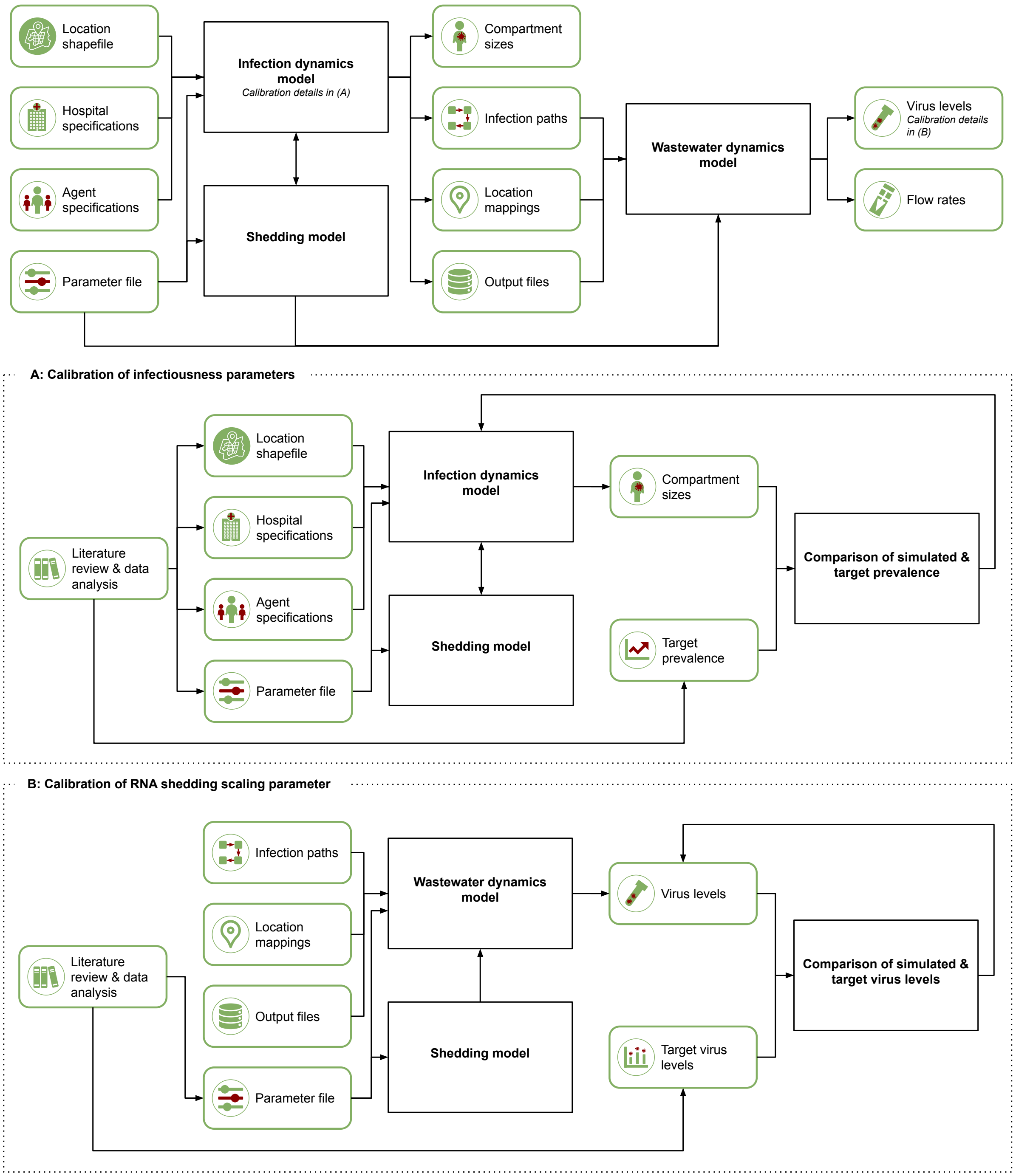

Supplementary Figure S2: Model workflow, related to Figures 2, 3, and 4. The flow of information from user inputs to the model components and ultimately to the final outputs. **(A)** Sub-workflow for calibrating the unknown infectiousness parameters within the infection dynamics model: the transmission scaling parameter κ, the initially exposed proportion e_{init}, the transmission damping level l_{damp}, and the damping start time t_{damp}. **(B)** Sub-workflow for calibrating the RNA shedding scaling parameter κ_{γ}.

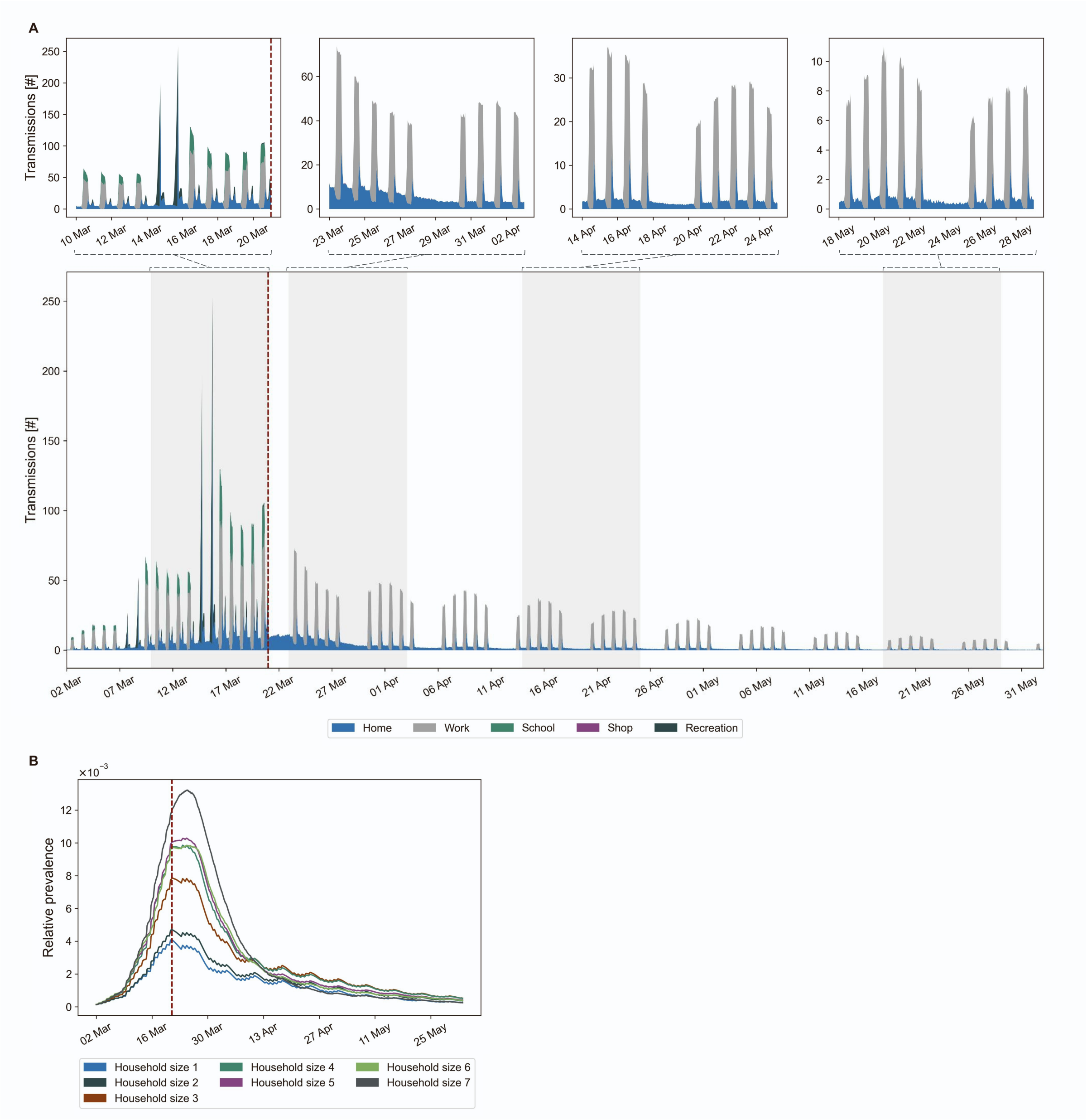

Supplementary Figure S3: Prevalence and transmission dynamics over time, related to Figures 2 and 4. **(A)** Mean number of transmissions over time for each location type, across 100 simulations. The red line indicates day 19 of the simulated time frame, when location closures begin. **(B)** Relative prevalence over time for every household size. Shown are the mean values across 100 simulations.

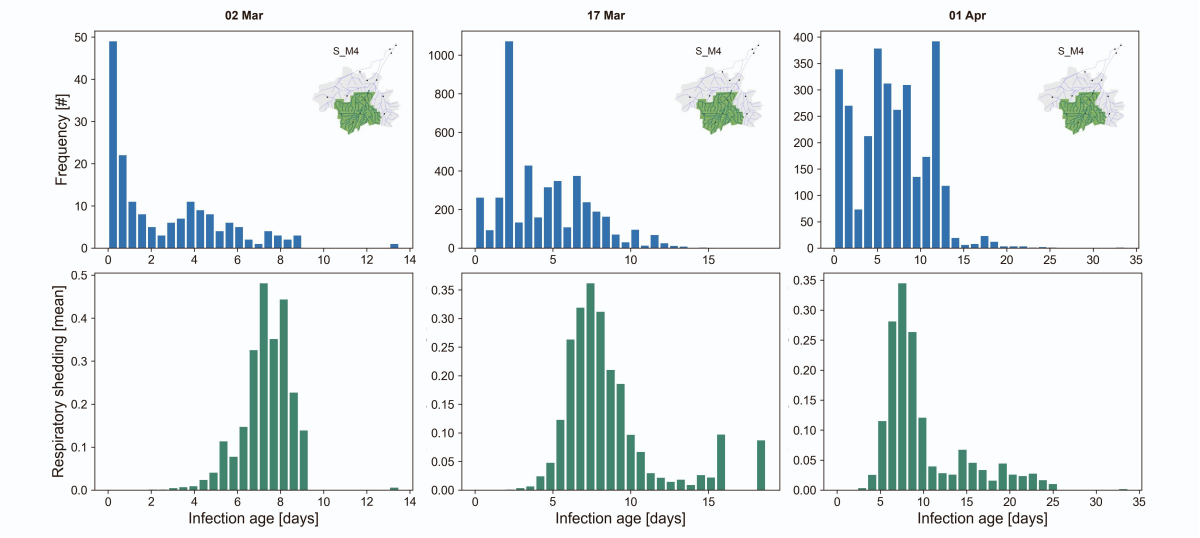

Supplementary Figure S4: Infection age and respiratory shedding upstream of station S_M4, related to Figure 2. Shown are the results of one simulation on three different days (02 Mar, 17 Mar, and 01 Apr) at midnight, when all agents are at *Home*. The first row shows the number of infected agents by infection age within the catchment area of sampling station S_M4. The second row shows the corresponding mean respiratory shedding value across infected agents with each given infection age.

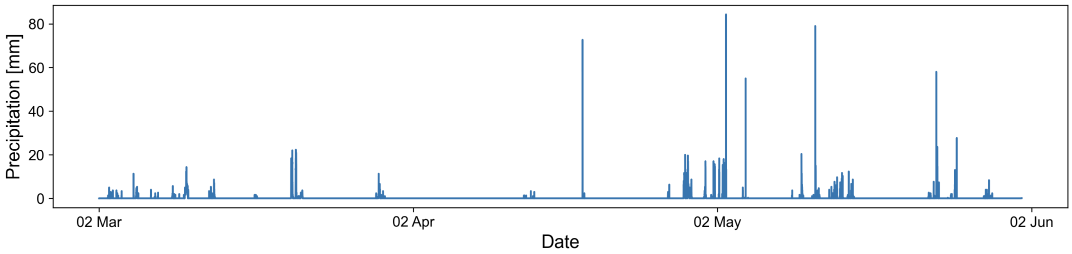

Supplementary Figure S5: Precipitation over time, related to Figures 3 and 5.

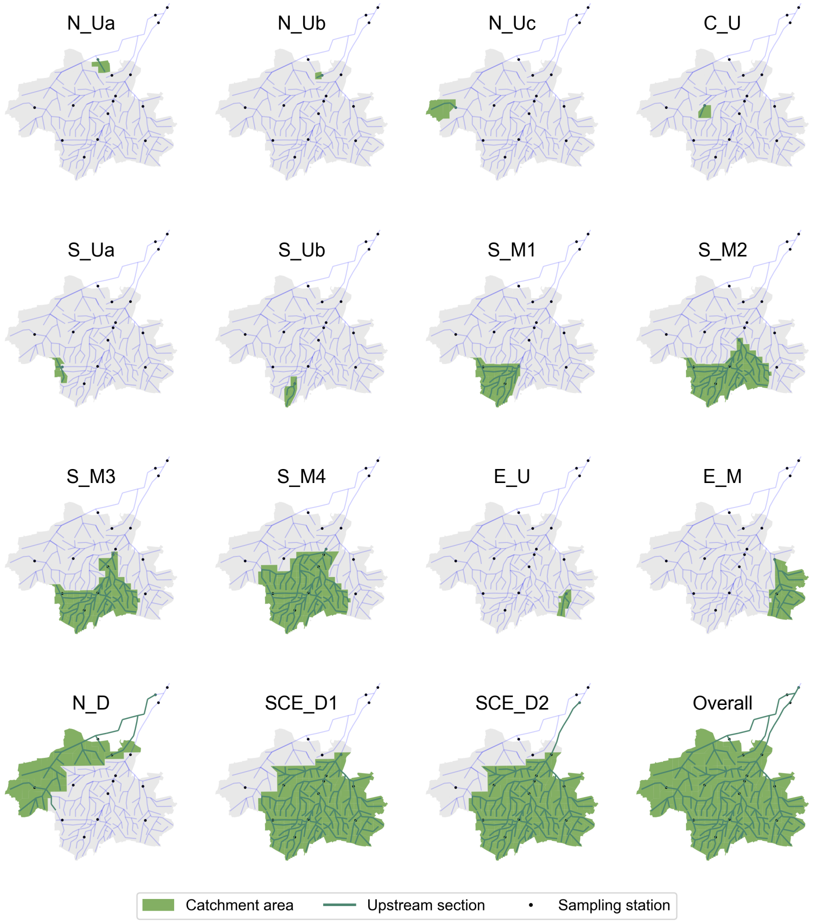

Supplementary Figure S6: Catchment areas per sampling location, related to Figure 3.

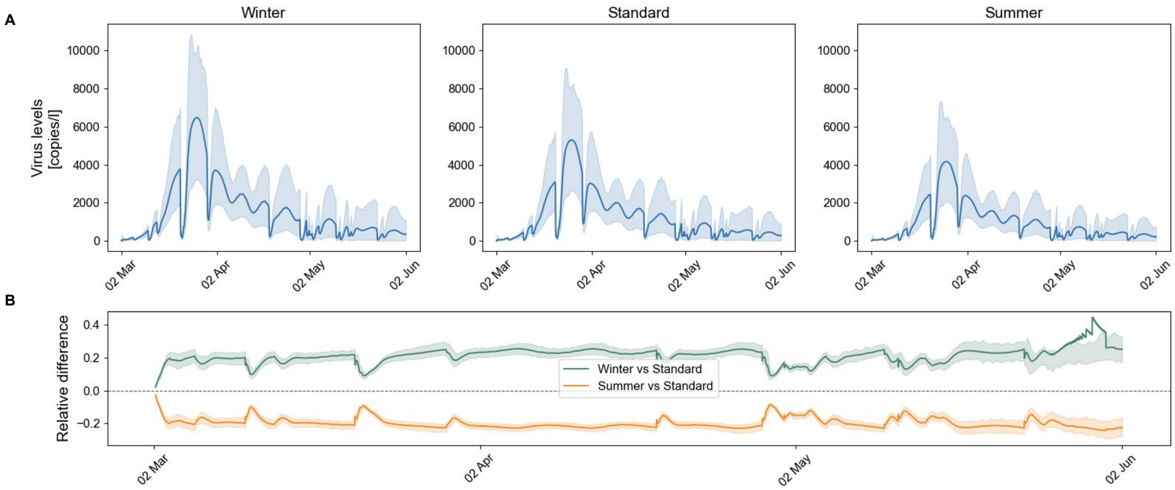

Supplementary Figure S7: Sensitivity to decay assumptions, related to Figure 3. (A) Virus levels for the sampling station N_D for three different decay scenarios: Winter ($k = 2.1 \cdot 10^{-6}$ per second), the standard setting used in this study representing a value typical for spring ($k = 4.7 \cdot 10^{-6}$ per second), and summer ($k = 8.1 \cdot 10^{-6}$ per second). The shaded area indicates the 90% simulation interval over 100 simulations. **(B)** Relative difference over time to the standard decay setting. The shaded area indicates the 90% simulation interval over 100 simulations.

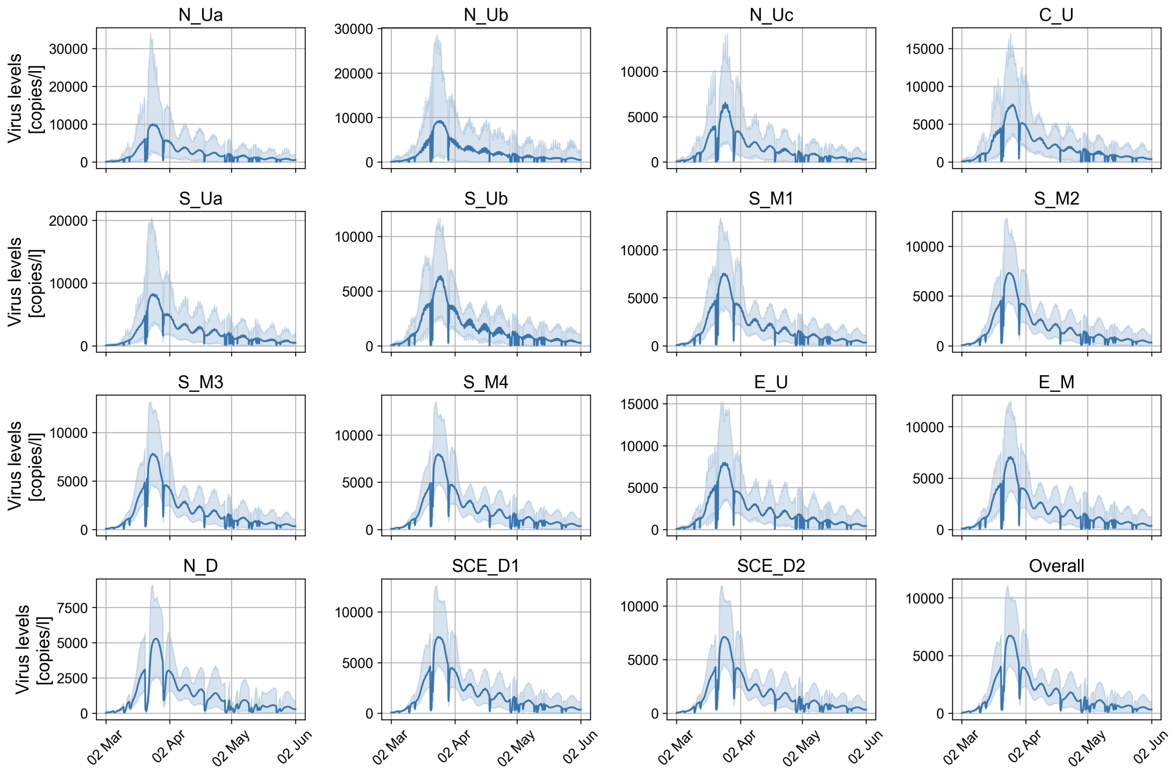

Supplementary Figure S8: Virus levels per sampling location for the decay & rain scenario (uniform initialization of prevalence), related to Figures 3, 5, 6, and 7. This corresponds to Scenario 3a in Supplementary Table S4 and reflects the standard experimental setup. The shaded area indicates the 90% simulation interval over 100 simulations.

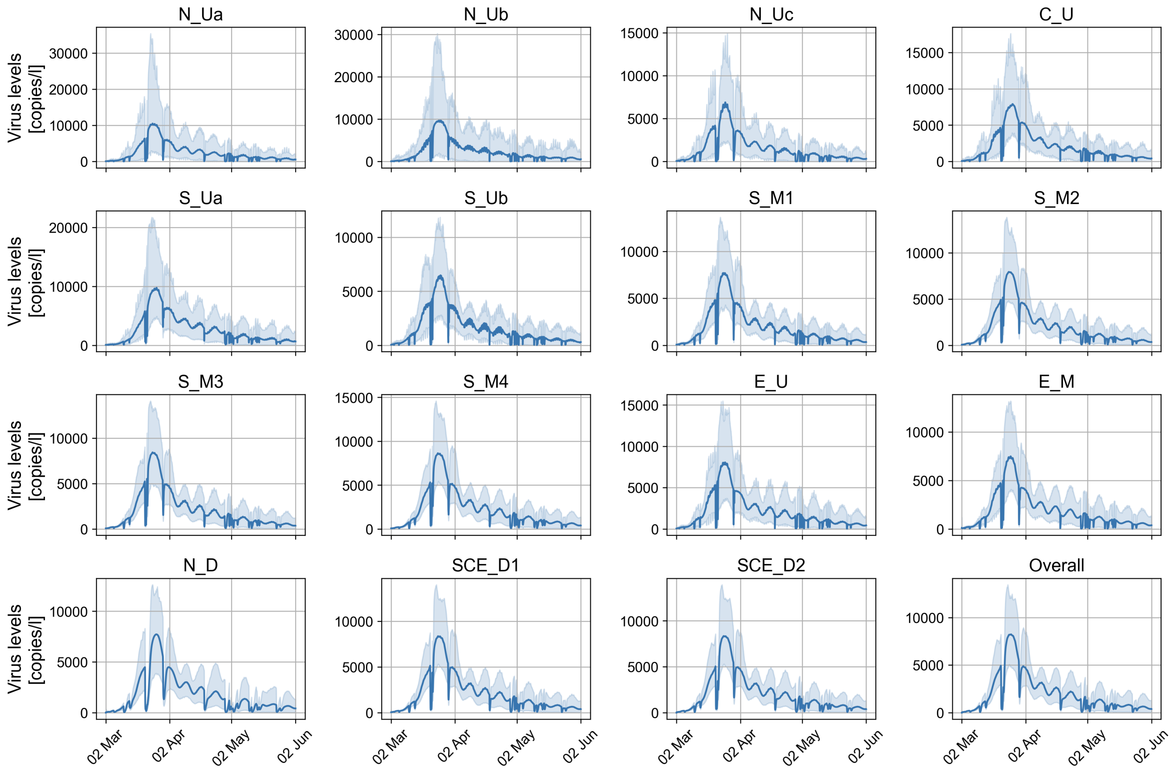

Supplementary Figure S9: Virus levels per sampling location for the no decay & rain scenario (uniform initialization of prevalence), related to Figure 3. This corresponds to Scenario 2 in Supplementary Table S4. The shaded area indicates the 90% simulation interval over 100 simulations.

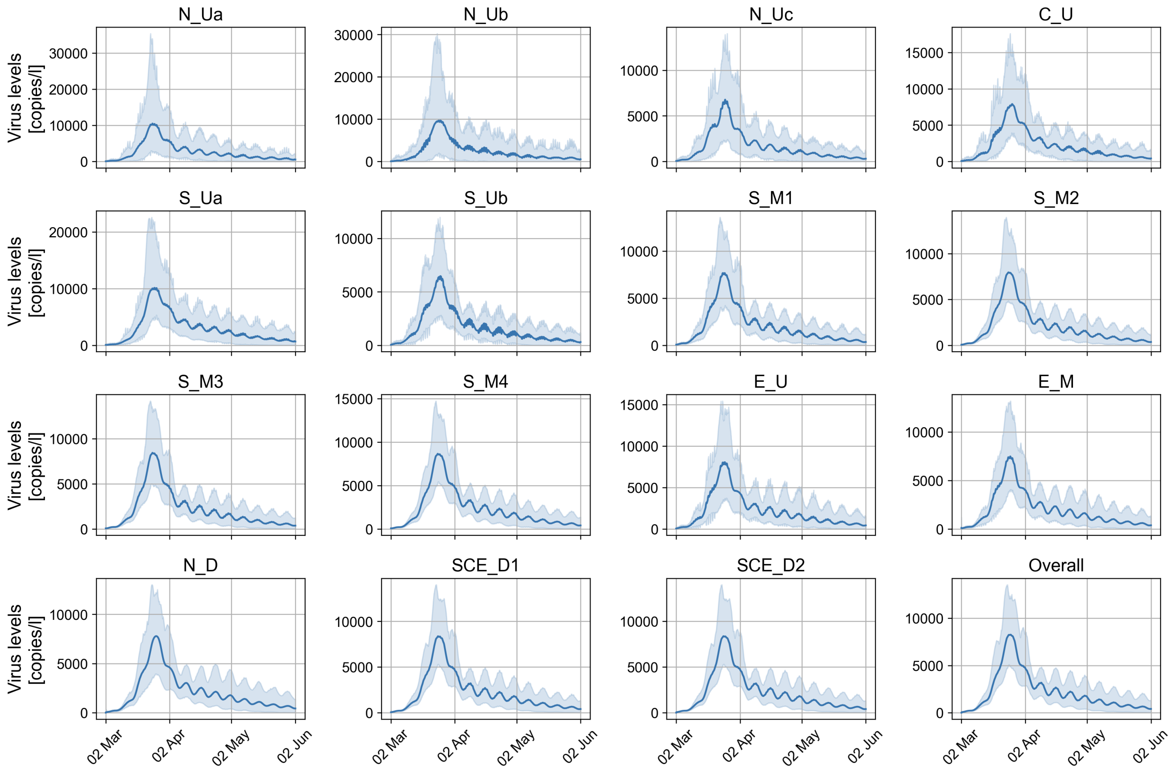

Supplementary Figure S10: Virus levels per sampling location for the no decay & no rain scenario (uniform initialization of prevalence), related to Figure 3 and 5. This corresponds to Scenario 1 in Supplementary Table S4. The shaded area indicates the 90% simulation interval over 100 simulations.

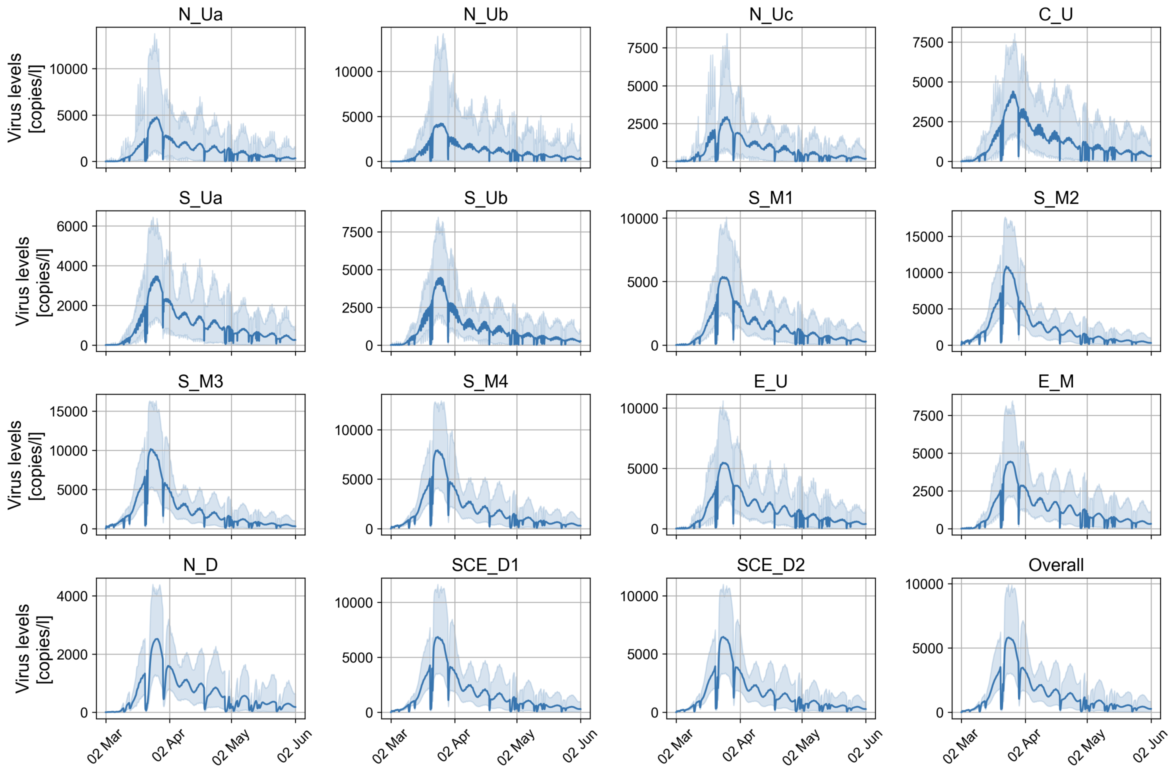

Supplementary Figure S11: Virus levels per sampling location for the decay & rain scenario (local initialization at the city center), related to Figures 3 and 6. This corresponds to Scenario 4 in Supplementary Table S4. The shaded area indicates the 90% simulation interval over 100 simulations.

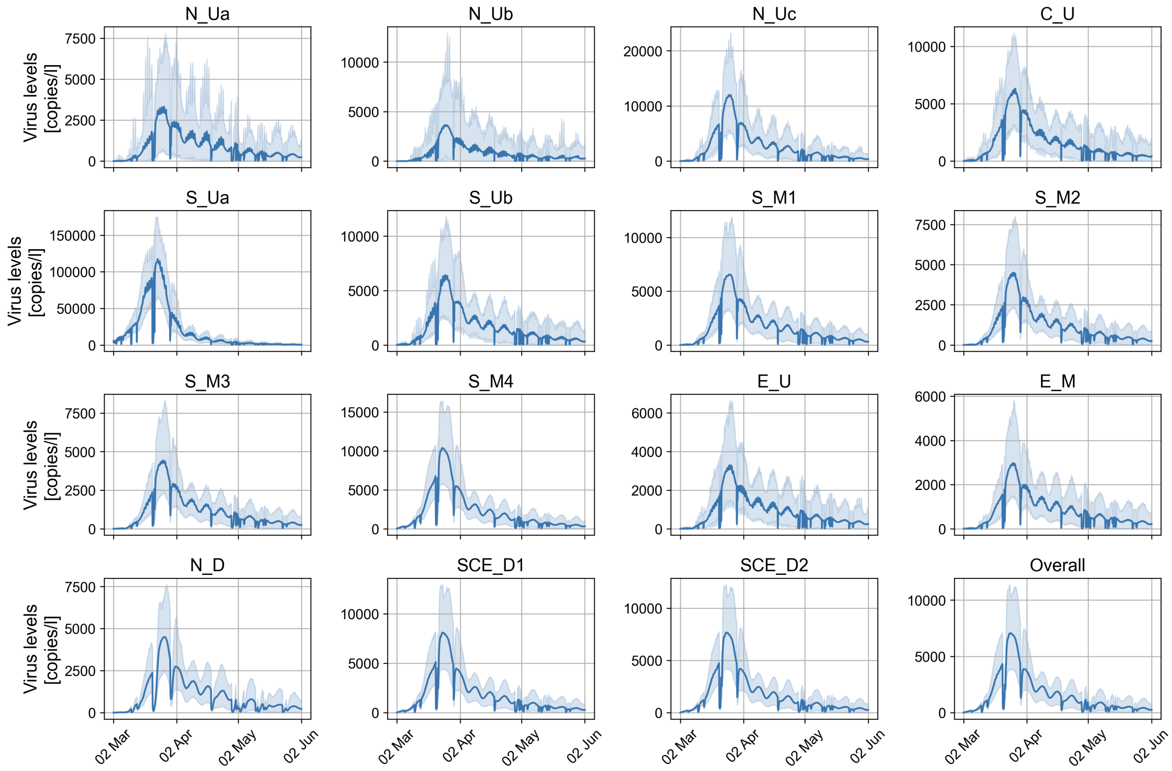

Supplementary Figure S12: Virus levels per sampling location for the decay & rain scenario (local initialization at the city border), related to Figures 3 and 6. This corresponds to Scenario 5 in Supplementary Table S4. The shaded area indicates the 90% simulation interval over 100 simulations.

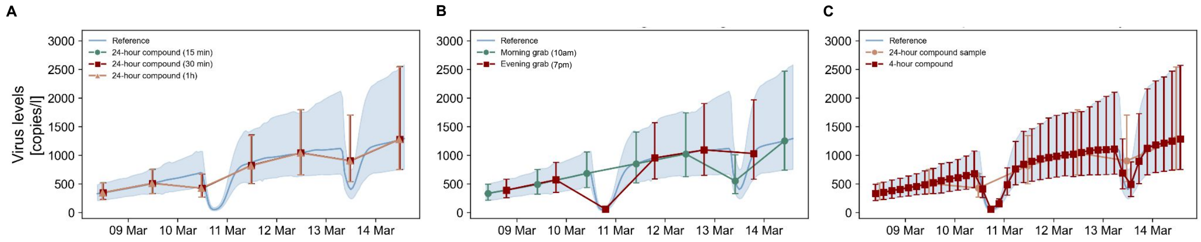

Supplementary Figure S13: Additional analysis of sampling methods at measurement station S_M2, related to Figure 5. (A) 24-hour compound sample using a resolution of 15 minutes (96 grabs within 24 hours), 30 minutes (48 grabs within 24 hours), or one hour (24 grabs within 24 hours). In all subplots, the shaded area indicates the 90% simulation interval over 100 simulations. **(B)** Comparison of morning vs. evening grab sampling. **(C)** 24-hour vs 4-hour compound sampling.

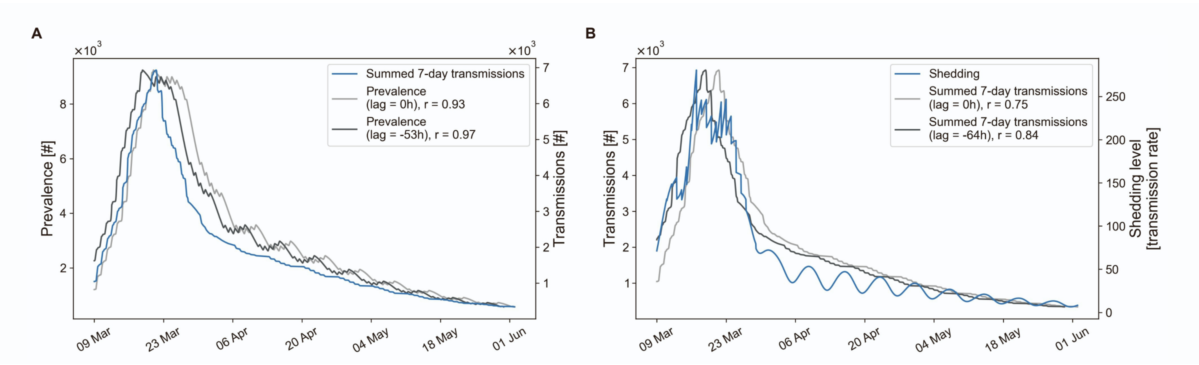

Supplementary Figure S14: Correlation of transmissions, prevalence and respiratory shedding, related to Figure 7. (A) Mean number of transmissions summed over the last 7 days and mean prevalence over time, across 100 simulations. The prevalence shifted by -51 hours has the highest correlation with the cumulative 7-day transmissions. In all subplots, the shaded area indicates the 90% simulation interval over 100 simulations. **(B)** Mean number of transmissions summed over the last 7 days and mean respiratory shedding level over time. The cumulative 7-day transmissions shifted by -66 hours has the highest correlation with the respiratory shedding level.

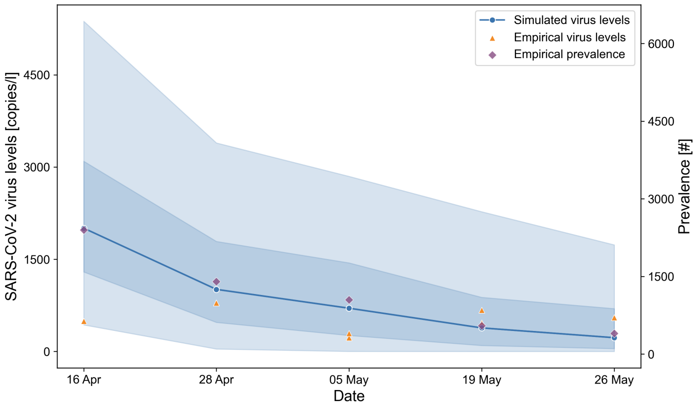

Supplementary Figure S15: Results for fitting of RNA shedding scaling factor, related to Figure 4. Comparison of the distribution of simulated and observed SARS-CoV-2 virus levels in wastewater across sampling locations in Munich (at each date for which at least one empirical measurement is available), based on the calibrated ABM parameters and a post-hoc estimated shedding scaling factor. The 90% interval, 50% interval, and median of the simulation results are shown. The estimated infection prevalence across Munich at each date is additionally shown in purple.

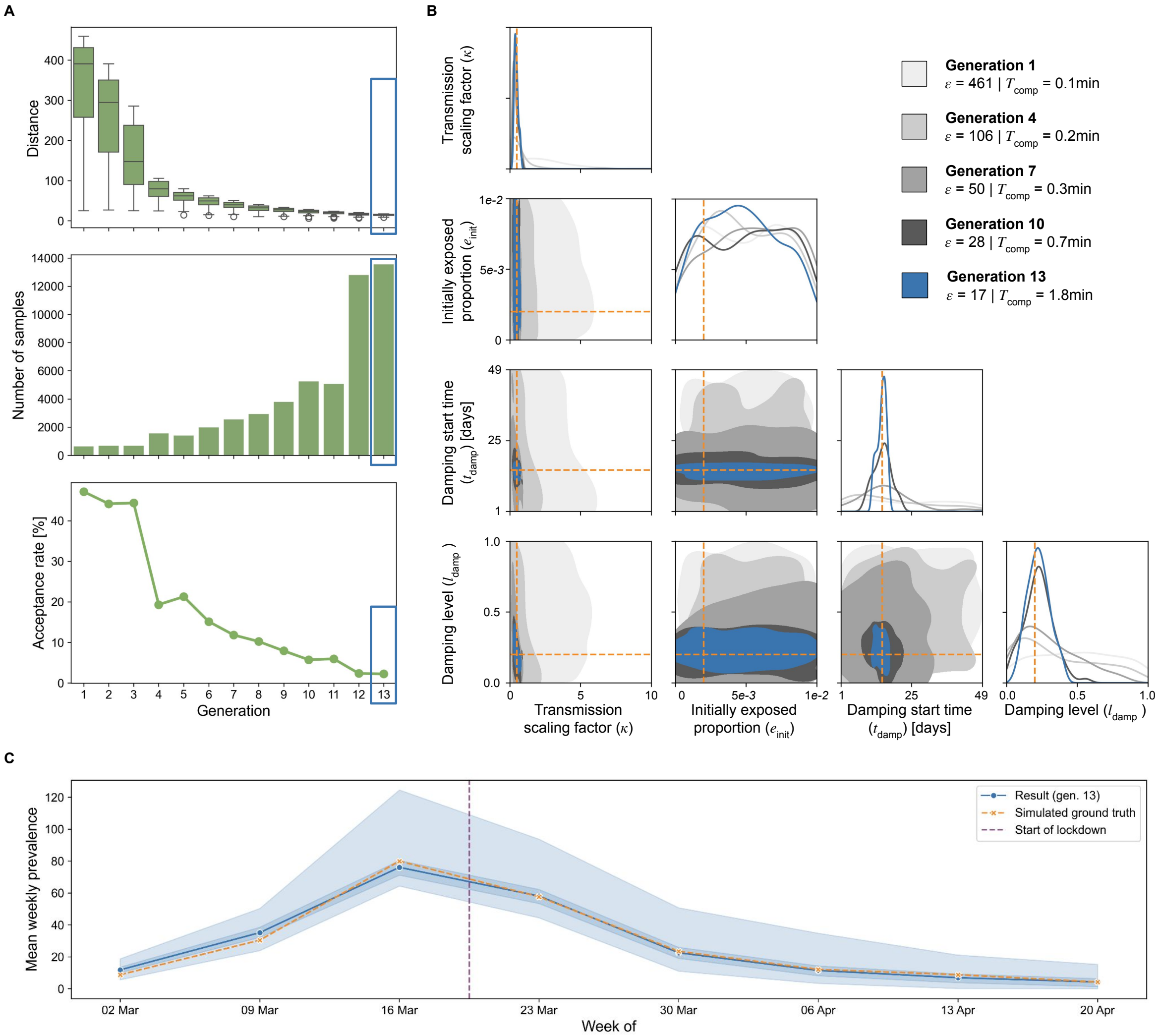

Supplementary Figure S16: Verification of fitting for small synthetic example, related to Figure 4. (A) Calibration diagnostics across generations: Euclidean distance between simulated and ground truth prevalence for accepted simulations; number of total samples; and acceptance rate. The generation used in subsequent analyses is outlined in blue. **(B)** Evolution of posterior parameter distributions, with the corresponding error values (ϵ) and computation times (T_{comp}). As in (A), the final accepted generation is in blue. Ground truth values are orange. **(C)** Agreement between simulated and ground truth mean weekly prevalence. The shaded blue areas indicate the 50% and 100% simulation intervals across the 300 accepted simulations from the final generation. As in (B), the ground truth is in orange.

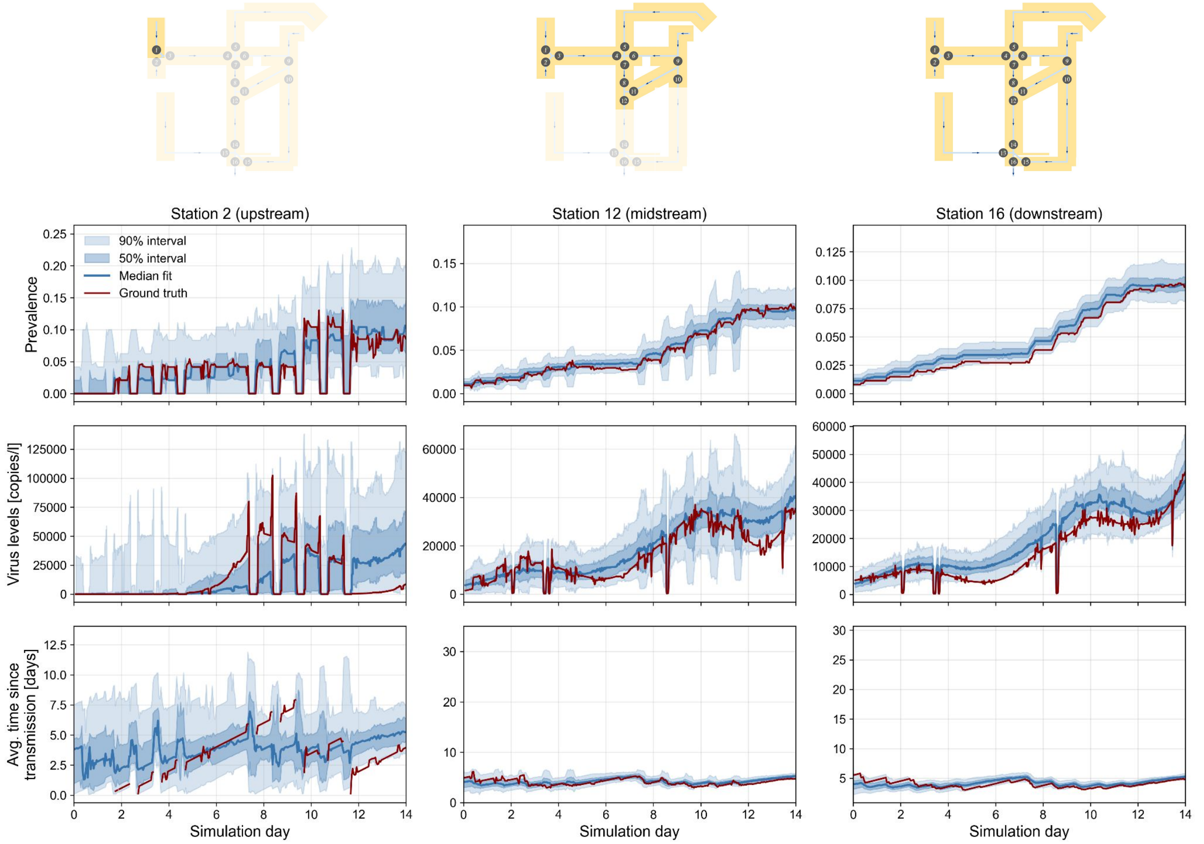

Supplementary Figure S17: Verification of viral load predictions, related to Figure 4. The catchment area of an upstream (left column), midstream (middle column), and downstream (right column) station are displayed in the top row. The subsequent rows show the proportional infection prevalence in the given catchment, the measured virus levels at the given station, and the average time since transmission across the infected agents in the given catchment. The average time since transmission is undefined when the upstream prevalence is zero, leading to gaps in its trajectory.

B Supplementary tables

Type	Number
DEMO trips	4,750,458
Agents obtained from DEMO trips	1,312,903
All ABM agents	1,536,985
All locations	1,043,855
Home locations	922,604
School locations	3,422
Work locations	63,674
Recreation locations	36,924
Shop locations	17,184
Hospital locations	30
ICU locations	17
Locations in catchment area	996,369

Supplementary Table S1: ABM Munich initialization details, related to Figure 2.

Task	Cores [#]	CPU specifics
Wastewater simulations	8	AMD Ryzen 9 5900HX
ABM scaling test	14	Intel Xeon “Skylake” Gold 6132
ABM fitting & simulations	48	2x AMD EPYC 7F72 3.20 GHz / 7443 2.85 GHz

Supplementary Table S2: Hardware specifics per computation task, related to Figures 2, 3, and 4.

Covariate	Uniform initialization		Local init. (city center) - full timeframe		Local init. (city center) - from 01 May		Local init. (city border) - full timeframe		Local init. (city border) - from 01 May	
	Mean coef.	% sig. sims	Mean coef.	% sig. sims	Mean coef.	% sig. sims	Mean coef.	% sig. sims	Mean coef.	% sig. sims
Mean *Recreation* size for residents	0.04	**89%**	0.11	**100%**	0.01	**52%**	0.11	**100%**	0.03	**56%**
Mean *Work* size for residents	0.02	**46%**	-0.07	76%	0.01	**32%**	0.01	0%	0.08	**83%**
No. of hospitals	0.02	34%	0.09	**99%**	0.01	25%	0.02	0%	0.04	36%
Working-age pop. proportion	0.03	25%	0.02	11%	0.01	16%	0.04	0%	-0.03	6%
Child population proportion	0.02	24%	0.07	17%	0.01	24%	-0.04	0%	0.03	16%
Mean *Shop* size for residents	0.01	21%	-0.05	23%	0.00	16%	0.10	**96%**	0.00	16%
Mean *Home* size	0.01	14%	-0.03	2%	0.00	13%	-0.01	0%	0.00	9%
Mean *School* size for residents	0.00	1%	-0.01	0%	0.00	13%	0.02	0%	0.00	5%

Supplementary Table S3: Predictors of infection risk across initialization scenarios, related to Figure 6. Results of per-simulation ordinary least squares regressions of area-level mean proportional prevalence against min-max normalized covariates. The table reports mean coefficients across 100 simulations per scenario and the percentage of those simulations with $p < 0.05$. Negative coefficients (protective effects) are in red, and the two most consistent predictors per scenario are in bold.

ID	MEmilio initialization	Rain scenario	Viral decay	Post-processing
1	Uniform	No precipitation	No	None
2	Uniform	Precipitation	No	None
3a	Uniform	Precipitation	Yes	None
3b	Uniform	Precipitation	Yes (standard)	24h compound (1h resolution)
3c	Uniform	Precipitation	Yes (winter)	24h compound (1h resolution)
3d	Uniform	Precipitation	Yes (summer)	24h compound (1h resolution)
3e	Uniform	Precipitation	Yes	4h compound (1h resolution)
3f	Uniform	Precipitation	Yes	24h compound (15min res.)
3g	Uniform	Precipitation	Yes	24h compound (30min res.)
3h	Uniform	Precipitation	Yes	Daily grab sampling (10am)
3i	Uniform	Precipitation	Yes	Daily grab sampling (7pm)
3j	Uniform	Precipitation	Yes	PMMoV normalization
3k	Uniform	Precipitation	Yes	Flow rate normalization
4	Local (city center)	Precipitation	Yes	None
5	Local (city border)	Precipitation	Yes	None

Supplementary Table S4: Overview of wastewater model scenarios implemented for the city of Munich, related to Figure 3.

Symbol	Description	Value (per age group)	Reference
$\mu_{I_{\rm ns}}^{I_{\rm sy}}$	Proportion of symptomatic cases per non-symptomatic case.	0.75 (0-15), 0.8 (16-80+)	[1]
$\mu_{I_{\rm sy}}^{I_{\rm sev}}$	Proportion of severe cases per symptomatic case.	0.0075 (0-15), 0.019 (16-34), 0.0615 (35-59), 0.165 (60-79), 0.225 (80+)	[1]
$\mu_{I_{\rm sev}}^{I_{\rm cri}}$	Proportion of critical cases per severe case.	0.075 (0-34), 0.15 (35-59), 0.3 (60-79), 0.4 (80+)	[1]
$\mu_{I_{\rm cri}}^{D}$	Proportion of dead cases per critical case.	0.05 (0-15), 0.14 (16-59), 0.4 (60-79), 0.6 (80+)	[1]
τ_E	Time (in days) spent in exposed state before transitioning to non-symptomatic state.	Lognormally distributed [2]; mean: 3.335 (All); variance: 0.1189 (All)	[2, 1]
$\tau_{I_{\rm ns}}^{I_{\rm sy}}$	Time (in days) spent in non-symptomatic state before transitioning to symptomatic state.	Lognormally distributed [2]; mean: 1.865 (All); variance: 0.1182 (All)	[2, 1]
$\tau_{I_{\rm ns}}^{R}$	Time (in days) spent in non-symptomatic state before transitioning to recovered state.	Lognormally distributed [2]; mean: 7 (All); variance: 0.4999 (All)	[2, 1]
$\tau_{I_{\rm sy}}^{I_{\rm sev}}$	Time (in days) spent in symptomatic state before transitioning to severe state.	Lognormally distributed [2]; mean: 10.5 (0-34), 6 (35-80+); variance: 0.5954 (0-34), 0.2662 (35-80+)	[2, 1]
$\tau_{I_{\rm sy}}^{R}$	Time (in days) spent in symptomatic state before transitioning to recovered state.	Lognormally distributed [2]; mean: 5 (0-15), 6 (16-34), 8 (35-59), 10 (60-79), 15 (80+); variance: 0.2689 (0-15), 0.2662 (16-34), 0.2636 (35-59), 0.2624 (60-79), 1.0562 (80+)	[2, 1]
$\tau_{I_{\rm sev}}^{I_{\rm cri}}$	Time (in days) spent in severe state before transitioning to critical state.	Lognormally distributed [2]; mean: 5 (All); variance: 1.1957 (All)	[2, 1]
$\tau_{I_{\rm sev}}^{R}$	Time (in days) spent in severe state before transitioning to recovered state.	Lognormally distributed [2]; mean: 7 (0-34), 17.5 (35-79), 12.5 (80+); variance: 1.1142 (0-34), 3.2941 (35-79), 1.6807 (80+)	[2, 1]
$\tau_{I_{\rm cri}}^{D}$	Time (in days) spent in critical state before transitioning to dead state.	Lognormally distributed [2]; mean: 6 (0-34), 16.5 (35-79), 11 (80+); variance: 1.1434 (0-34), 0.5896 (35-79), 0.262 (80+)	[2, 1]
$\tau_{I_{\rm cri}}^{R}$	Time (in days) spent in critical state before transitioning to recovered state.	Lognormally distributed [2]; mean: 7 (0-34), 17.5 (35-79), 12.5 (80+); variance: 1.1142 (0-34), 3.2941 (35-79), 1.6807 (80+)	[2, 1]
$v_{\rm max}^{\rm sy}$	Peak viral load value in $\log_{10}$ units for symptomatic infections.	8.1	[3, 2]
a	Shedding function parameter.	-7	[3, 2]
b	Shedding function parameter.	1	[3, 2]
κ	Scaling factor translating the shedding rate to a transmission rate.	Maximum a posteriori estimate (MAP): 0.66; min: 0.39; max: 1.77	calibrated
κ_γ	Scaling factor translating the shedding rate to an RNA shedding rate.	$7.56 \cdot 10^8$	calibrated
k	Viral decay rate for viral load in sewage.	$4.7 \cdot 10^{-6}$ (standard setting), $2.1 \cdot 10^{-6}$ (winter), $8.1 \cdot 10^{-6}$ (summer) per second	[4]
n_{school}^{max}	Maximum size of school locations.	45	[5]
n_{work}^{max}	Maximum size of work locations.	40	[5]
$t_{\rm damp}$	Time point of transmission rate damping.	MAP: Day 13; min: Day 9; max: Day 27	calibrated
$l_{\rm damp}$	Transmission rate damping factor.	MAP: 0.11; min: 0.07; max: 0.34	calibrated
$t_{closure}^{work}$	Time point of *Work* location closure.	Day 19 (21 March 2020)	[6]
$\mu_{closure}^{work}$	Proportion of *Work* locations closed.	0.37	[7]
$t_{closure}^{school}$	Time point of *School* location closure.	Day 14 (16 March 2020)	[6]
$\mu_{closure}^{school}$	Proportion of *School* locations closed.	1.0	[6]
$t_{closure}^{recreation}$	Time point of *Recreation* location closure.	Day 19 (21 March 2020)	[6]
$\mu_{closure}^{recreation}$	Proportion of *Recreation* locations closed.	0.51	[7]
$t_{closure}^{shop}$	Time point of *Shop* location closure.	Day 19 (21 March 2020)	[6]
$\mu_{closure}^{shop}$	Proportion of *Shop* locations closed.	0.13	[7]
$E^{(0)}$	Proportion of initially exposed agents.	MAP: 0.000099; Min: 0.000006; Max: 0.000129	calibrated
$I_{ns}^{(0)}$	Proportion of initially non-symptomatic agents.	0.00005	[8]
$I_{sy}^{(0)}$	Proportion of initially symptomatic agents.	0.00002	[8]

Supplementary Table S5: Infection dynamics and shedding model parameters, related to Figure 2.

References

[1] Martin J. Kühn, Daniel Abele, Tanmay Mitra, Wadim Koslow, Majid Abedi, Kathrin Rack, Martin Siggel, Sahamoddin Khailaie, Margrit Klitz, Sebastian Binder, Luca Spataro, Jonas Gilg, Jan Kleinert, Matthias Häberle, Lena Plötzke, Christoph D. Spinner, Melanie Stecher, Xiao Xiang Zhu, Achim Basermann, and Michael Meyer-Hermann. Assessment of effective mitigation and prediction of the spread of SARS-CoV-2 in Germany using demographic information and spatial resolution. *Mathematical Biosciences*, 339:108648, 2022.

[2] David Kerkmann, Sascha Korf, Khoa Nguyen, Daniel Abele, Alain Schengen, Carlotta Gerstein, Jens Henrik Göbbert, Achim Basermann, Martin J. Kühn, and Michael Meyer-Hermann. Agent-based modeling for realistic reproduction of human mobility and contact behavior to evaluate test and isolation strategies in epidemic infectious disease spread. *Computers in Biology and Medicine*, 193:110269, 2025.

[3] Terry C. Jones, Guido Biele, Barbara Mühlemann, Talitha Veith, Julia Schneider, Jörn Beheim-Schwarzbach, Tobias Bleicker, Julia Tesch, Marie Luisa Schmidt, Leif Erik Sander, Florian Kurth, Peter Menzel, Rolf Schwarzer, Marta Zuchowski, Jörg Hofmann, Andi Krumbholz, Angela Stein, Anke Edelmann, Victor Max Corman, and Christian Drosten. Estimating infectiousness throughout SARS-CoV-2 infection course. *Science*, 373(6551):eabi5273, 2021.

[4] Jean-Baptiste Burnet, Henry-Michel Cauchie, Cécile Walczak, Nathalie Goeders, and Leslie Ogorzaly. Persistence of endogenous RNA biomarkers of SARS-CoV-2 and PMMoV in raw wastewater: Impact of temperature and implications for wastewater-based epidemiology. *Science of The Total Environment*, 857 (Part 2):159401, 2023.

[5] Joël Mossong, Niel Hens, Mark Jit, Philippe Beutels, Kari Auranen, Rafael Mikolajczyk, Marco Massari, Stefania Salmaso, Gianpaolo Scalia Tomba, Jacco Wallinga, Janneke Heijne, Malgorzata Sadkowska-Todys, Magdalena Rosinska, and W. John Edmunds. Social contacts and mixing patterns relevant to the spread of infectious diseases. *PLoS Medicine*, 5(3):e74, 2008.

[6] A3M Global Monitoring GmbH. COVID-19 pandemic - Germany. `https://global-monitoring.com/gm/page/events/epidemic-0001937.eP207vO5rIwe.html`, 2025. `https://global-monitoring.com/gm/page/events/epidemic-0001937.eP207vO5rIwe.html`, accessed: 02 Apr 2025.

[7] Google LLC. Google COVID-19 Community Mobility Reports. `https://www.google.com/covid19/mobility/`, 2024. `https://www.google.com/covid19/mobility/`. Accessed: 01 Mar 2024.

[8] Lorenzo Contento, Noemi Castelletti, Elba Raimúndez, Ronan Le Gleut, Yannik Schälte, Paul Stapor, Ludwig Christian Hinske, Michael Hoelscher, Andreas Wieser, Katja Radon, Christiane Fuchs, Jan Hasenauer, and the KoCo19 study group. Integrative modelling of reported case numbers and seroprevalence reveals time-dependent test efficiency and infectious contacts. *Epidemics*, 43:100681, 2023.
